# Different Types of Gel Carriers as Metronidazole Delivery Systems to the Oral Mucosa

**DOI:** 10.3390/polym12030680

**Published:** 2020-03-19

**Authors:** Magdalena Wróblewska, Emilia Szymańska, Marta Szekalska, Katarzyna Winnicka

**Affiliations:** Department of Pharmaceutical Technology, Faculty of Pharmacy, Medical University of Białystok, A. Mickiewicza 2C, 15-222 Białystok, Poland; esz@umb.edu.pl (E.S.); marta.szekalska@umb.edu.pl (M.S.); kwin@umb.edu.pl (K.W.)

**Keywords:** bioadhesion, metronidazole, hydrogel, bigel, oleogel, ex vivo permeation

## Abstract

Periodontal diseases are some of the most widespread oral afflictions, and they are labeled as chronic infections caused by the accumulation of bacteria in dental plaque that produces localized inflammation of the periodontium. The use of local drug delivery systems to treat periodontal diseases has received greater attention, because the active substance is targeted directly to the affected area, which minimizes its systemic side effects. Therefore, the purpose of the investigation was to develop and characterize different types of gel formulations—bigel, hydrogel and oleogel—as local delivery systems containing metronidazole (MET), which can be applied to the oral mucosa. The influence of the formulation type on the mechanical, rheological and mucoadhesive properties were examined. Moreover, in vitro release of metronidazole, its ex vivo permeation through buccal porcine mucosa and antimicrobial activity measured by the plate diffusion method were estimated. It was found that the gel formulations obtained were non-Newtonian systems, showing a shear-thinning behavior and thixotropic properties with good textural features such as firmness, compressibility and adhesiveness. Moreover, the preparations designed possessed beneficial mucoadhesive properties. The formulated hydrogels and bigels containing micronized MET were considered as better formulations in terms of drug release and antimicrobial activity compared to commercially available metronidazole ointment. An ex vivo permeation study with the use of porcine buccal mucosa demonstrated that the bigel formulation was characterized by higher initial permeability rate providing a fast therapeutic effect with simultaneous moderate retention in mucosal tissue to decrease the risk of local cytotoxicity.

## 1. Introduction

Periodontal diseases are very common oral afflictions that may affect as much as 90% of the worldwide population [1]. They are defined as a chronic inflammation of gum and tissues that surround and support the teeth and are classified into two major stages according to the severity of the disease, namely gingivitis and periodontitis. Gingivitis, the mildest form and an early stage of periodontal disease, is a nondestructive periodontal infection caused by the bacterial biofilm that accumulates on teeth adjacent to the gingival. Periodontitis, an advanced stage characterized by progression of gingivitis, may even lead to loss of teeth and erosion of supporting tissues. Furthermore, periodontitis may become more complicated and dangerous when the microbes from the infected gums penetrate the blood stream and develop systemic disease such as rheumatoid arthritis and cardiovascular disease [2,3]. It is well recognized that periodontal diseases are bacterial in nature. Numerous studies have shown that the bacterial flora in the periodontal infection area are polymicrobial; both Gram-positive and Gram-negative bacteria tend to aggregate and coexist in dental plaque [4,5]. The primary aim of periodontal disease treatment is to eliminate or control these pathogens. Different strategies are commonly used to treat periodontitis such as enhancement of patient hygiene, scaling, antibiotic administration or even surgical procedures [6]. Alternatively, local drug delivery systems against periodontal pathogens have been promoted due to the disadvantages of systemic administration as well as growing antibiotic resistance.

Many local drug delivery systems such as implants, films, fibers, ointments or gels prepared using biodegradable or nondegradable polymers have been tested for periodontal therapy. Among these carriers, mucoadhesive gel formulations have received considerable attention in treating periodontitis [7,8,9,10]. Generally, gel-based preparations are categorized into two main groups based on the polarity of the external liquid phase. In hydrogels, water is the external liquid phase, whereas oil is the external liquid phase of oleogels/organogels [11]. Hydrogels are formed by the three-dimensional network of either natural or synthetic gelling agents to immobilize the aqueous phase. These formulations ensure better patient compliance because of their specific properties such as easy removal after application, cooling effect, a greaseless texture and good spreadability. Hydrogels exhibit high biocompatibility and mucoadhesive properties, as they can adhere to the mucosa in the dental pocket and reduce irritation at the site of application. A disadvantage of using hydrogels is that they may be inadequate vehicles for lipophilic drugs [12,13]. Oleogels, also named organogels, are defined as semi-solid preparations obtained by gelation of lipophilic liquids with the use of suitable substances referred to as organogelators. The gelling agent forms aggregates and linkages between aggregates, which results in the formation of three-dimensional networks. Oleogels possess favorable rheological properties, ensure better solubility of hydrophobic compounds and, due to the absence of water, are resistant to microbial contamination and do not require the addition of preservatives. Moreover, in periodontal therapy, using lipid carriers may provide prolonged liberation, protect active substances from degradation in hydrophilic media and mask their unpleasant taste [7,14]. However, oleogels are considered to be oily in nature; thus, bigels were introduced as an alternative gel system. Bigels are biphasic systems produced by mixing hydrogel with oleogel at high rotation rates, resulting in a stable structured system. They have the advantages of both hydrogels and oleogels, such as ease of preparation and washing with water, good spreadability, satisfactory stability at room temperature, cooling effects and lower risk of irritation due to the absence of emulsifiers at high concentration. In addition, bigels are considered as good drug carriers for both lipophilic and hydrophilic active compounds [7,15].

Antibiotics and antiseptics have been commonly used to treat moderate to severe periodontal diseases [4,6]. Among a number of active compounds, metronidazole (MET) is regarded as a highly effective antimicrobial agent in treating adult periodontal disease [16,17,18]. MET, which is (1-(β-hydroxyethyl)-2-methyl-5-nitroimidazole), a synthetic nitroimidazole derivative, is used as an antibacterial agent with added antiparasitic properties. It is generally very well tolerated, and it has a wide therapeutic index. MET possesses poor water solubility and hydrophobicity. MET’s solubility in water was reported as 10.5 mg/mL at 25 °C [19]. Owing to a low logP value (−0.18), MET is classified as a hydrophilic drug [20]. It is available in a variety of drug forms, such as capsules, tablets suppository preparations and topical formulations, which are used for the treatment of different infections [20,21].

The purpose of this study was to enhance local delivery of MET using three types of gel formulations, namely hydrogel, oleogel and bigel, to cure periodontal diseases. The physicochemical, rheological, textural and bioadhesive properties of the designed gels were studied. Moreover, the antimicrobial activity of prepared gel carriers was estimated. Furthermore, in vitro MET release and its ex vivo permeation were examined to select the optimal gel formulation that may be used as the most effective local drug delivery system applied to the oral mucosa.

## 2. Materials and Methods

### 2.1. Materials

Metronidazole (MET) and glycerol 85% were purchased from PPH Galfarm Sp. z.o.o (Kraków, Poland). Aerosil^®^ 200 (colloidal silicon dioxide, fumed silica) was a gift from Evonik Industries AG, Hanau, Germany. Tocopheryl acetate (vitamin E), linseed oil (Pharma Cosmetics, Kraków, Poland), methyl paraben and propyl paraben (POL-AURA, Olsztyn, Poland), Tween 80 (Sigma Aldrich, Madrid, Spain), low viscosity sodium alginate (with viscosity of 132.6 mPa·s of 2% solution at 25 °C; molecular weight about 147,000), 61% mannuronic acid and 39% guluronic acid (Sigma Aldrich, Steinheim, Germany), sodium azide (Sigma Aldrich, Saint Louis, MO, USA), sodium chloride, sodium hydroxide potassium, potassium phosphate monobasic, sodium phosphate dibasic, methanol (Chempur, Piekary Śląskie, Poland), ethanol 99.9% (J.T. Baker, Deventer, Holland), acetonitrile HPLC grade and ethyl alcohol 96% (POCH, Gliwice, Poland) were used as received. All chemicals and solvents used for the study were of analytical grade. Simulated saliva solution (SSS, pH 6.8) was prepared by dissolving 8 g sodium chloride, 0.19 g potassium phosphate monobasic and 2.38 g of sodium phosphate dibasic in 1 L of water. Mueller–Hinton agar was provided by Biomaxima (Lublin, Poland). Cuprophan^®^ was received from Medicell (London, UK). Metronidazol 100 mg/g ointment with a composition of PEG 400 Da, PEG 4000 Da and cetostearyl alcohol used as a control was a product of CHEMA-ELEKTROMET (Rzeszów, Poland). The control strains *Staphylococcus aureus* ATCC 29213 and *Escherichia coli* ATCC 25922 were from TCS Biosciences Ltd., Buckingham, United Kingdom. Cellulose acetate membrane filters (CA 0.45 μm) were received from Millipore (Billerica, MA, USA). Buccal porcine mucosa was derived from a local veterinary service. This procedure did not require the approval of the local ethical committee on animal testing. Samples of the mucosa were stored at −20 °C and before the experiment were defrosted and cut into 5 mm diameter pieces.

### 2.2. Preparation and Characterization of MET

#### 2.2.1. Micronization of MET

Milling of MET for particles size reduction was performed in an oscillating ball mill MM 400 (Retsh^®^ GmbH, Haan, Germany) testing different conditions; time from 5 to 60 min and at rotational speed of 20 Hz 1 g of powder with three ZrO_2_ balls having diameter of 7 mm were charged in a cylindrical jar of the mill to fill one third of the volume of the jar. For the best process conditions of metronidazole micronization (MMET), 30 min and 20 Hz were chosen.

#### 2.2.2. DSC Analysis

Measurements were performed using an automatic thermal analyzer system (DSC TEQ2000, TA Instruments, New Castle, DE, USA). Samples of MET and MMET (in the amount of 5 mg) were placed in aluminum pans and heated in the range 25–300 °C under a 20 mL/min nitrogen flow [22].

#### 2.2.3. Solubility Studies

The solubility of MET and MMET was determined by using the shake-flask method. Briefly, an excess amount of MET/MMET was added to each vial containing 5 mL of the selected solvents (water, phosphate buffer 6.8, linseed oil). Mixtures were mechanically shaken for 24 h at 25 ± 0.5 °C and allowed to stand for 24 h to attain equilibrium. Then mixtures were centrifuged at 3000 rpm for 15 min, followed by filtration through a CA membrane filter (0.45 μm), diluted appropriately with methanol and analyzed by HPLC method at 319 nm against a standard [22,23].

### 2.3. Preparation and Physicochemical Characteristics of Hydrogels, Oleogels and Bigels

#### 2.3.1. Preparation of Hydrogels, Oleogels and Bigels

Different types of gel systems, namely hydrogel, oleogel and bigel, were prepared using an RZR 2020 mechanical stirrer (Heidolph Instruments, Schabach, Germany). The compositions of the designed formulations are listed in Table 1.

Alginate hydrogel (H) was prepared by gradually dispersing gelling agent (sodium alginate, 1.75% *w*/*w*) in an aqueous-based solution containing glycerol 85% (as a humectant) and parabens, previously dissolved in 96% ethanol, with the help of mechanical stirrer at a moderate speed. Mixing was continued until transparent hydrogels were received.

For the preparation of oleogel (O), Tween 80 was added to the heated oil (70 °C) with constant mechanical stirring at 300 rpm, and next the gelling agent Aerosil^®^ 200 was dispersed in a mixture. After complete blending of gelling agent, tocopheryl acetate as an antioxidant was added to the formulation. Once the mixture was homogenous, heating was stopped, and it was cooled down to the room temperature as it gradually solidified to form an oleogel.

Bigel (B) was developed by separately forming oleogel and hydrogel and then mixing them using a mechanical stirrer (800 rpm) at room temperature. Bigel formulations were made at different hydrogel to oleogel weight ratios, namely 30:70, 50:50 and 70:30. Stable and homogeneous bigel was obtained only using the hydrogel to oleogel weight ratio of 70:30.

MET or MMET (micronized metronidazole) at 10.0% *w*/*w* concentration was uniformly dispersed in gel vehicles. The concentration of active ingredient was set based on commercially available product. Control gel formulations without MET (placebo) were also initially prepared.

#### 2.3.2. Drug Content Analysis by HPLC Method

MET content was determined after extraction of gel formulation samples in ethanol (99.9%) and analyzed by HPLC method using an Agilent Technologies 1200 HPLC system equipped with a G1312A binary pump, a G1316A thermostat, a G1379B degasser and a G1315B diode array detector (Agilent, Waldbronn, Germany) in the following conditions: Zorbax Eclipse XDB-C18, 4.6 × 150 mm, 5 µm column (Agilent, Waldbronn, Germany); mobile phase: acetonitrile—0.01M phosphate buffer pH 4.7 (15:85, *v*/*v*); flow rate 1.0 mL/min; detection at 319 nm; retention time 3.0 min [22]; the standard calibration curve was linear over the range of 2.5–150 µg/mL (R^2^ = 0.999).

#### 2.3.3. pH Determination

The pH was measured by a glass electrode of the pH-meter Orion 3 Star (Thermo Scientific, Waltham, MA, USA), which was calibrated before each use with standard buffer solutions. Each measurement was carried out three times and average pH was calculated.

#### 2.3.4. Particle Size Analysis

Gel formulation samples (in quantity corresponding to 10 μg of MET/MMET) were observed under 100× magnification, and particles size was analyzed by using a Motic BA 400 optical microscope equipped with a camera (Moticon, Wetzlar, Germany) [24].

#### 2.3.5. Viscosity Measurement and Determination of Rheological Properties

The viscosity was determined using a Brookfield viscometer (RVDV-III Ultra, Brookfield Engineering Laboratories, Middlebro, MA, USA) equipped with a cone/plate type CPA52Z (plate diameter 24 mm, cone angle 3°) measuring system, at a temperature of 25 ± 1 °C. The viscosity values of gel formulations at a shear rate of 10.00 s^−1^ were recorded, and the rheograms were evaluated by plotting the obtained values of shear stress versus shear rate (2.00–20.00 s^−1^) [25].

#### 2.3.6. Texture Analysis

Texture properties of prepared hydrogel, oleogel and bigel formulations were examined using a TA.XT Plus texture analyzer (Stable Micro System, Godalming, UK) for backwards extrusion measurements. A disc (35 mm diameter) was pushed at a speed of 2 mm·s^−1^ for a distance of 5 mm into the sample (30 g) and redrawn. Data collection and data analysis were performed using the Texture Exponent software package. Parameters such as firmness, compressibility and adhesiveness were determined from the force–time plots [25,26,27].

### 2.4. Ex Vivo Bioadhesive Properties

Evaluation of bioadhesiveness was performed using a TA.XT Plus texture analyzer (Stable Micro Systems, Godalming, UK) on the porcine buccal mucosa model. Samples of the mucosa were frozen at −20 °C and stored no longer than 4 weeks. On the day of the experiment, mucosa was defrosted and cut into 5 mm diameter pieces. Next, samples were thawed in physiological saline solution (0.9% NaCl) at 25 ± 0.5 °C for 30 min; mucosa was attached to the lower end of a cylindrical probe using a cyanoacrylate glue, and gel formulations in the amount of 0.5 g were placed below the probe. The experiment was carried out at 37 ± 0.5 °C (water bath) to mimic in vivo conditions. Experimental parameters of the process were chosen during preliminary tests and set as follows: pre-test speed 0.5 mm∙s^−1^, test speed 0.1 mm∙s^−1^, contact time 120 s, post-test 0.1 mm∙s^−1^, applied force 0.5 N. The adhesive properties were determined as the maximum detachment force (F_max_), and the work of adhesion (W_ad_) was calculated from the area under the force versus distance curve, expressed in µJ. The work of adhesion (W_ad_) was calculated using the following formula:

W_ad_ = A × 0.1 × 1000

where A is area under the force versus distance curve, the multiplication by 0.1 is the conversion time measurement to distance (the sampler was raised at 0.1 mm·s^−1^) and the multiplication by 1000 is in order to express the result in units of work (µJ) [27,28].

### 2.5. In Vitro Release

The release of MET/MMET was measured through a cellulose membrane (Cuprophan^®^, Medicell, London, UK) using an enhancer cell with a surface area of 3.80 cm^2^. The enhancer cell consisted of a Teflon load ring, a cap, a membrane and a drug reservoir. This study was performed using the USP dissolution apparatus 2 (Agilent 708-DS, Agilent Technologies, Cary, NC, USA) with mini vessels (250 mL) and mini paddles. Samples, each of about 3 g, were placed in the enhancer cell, which was then immersed in the dissolution vessel containing 100 mL of the release medium–simulated saliva solution (SSS), pH 6.8 (the sink conditions were maintained), previously warmed to 37 ± 0.5 °C. Agitation was provided by mini paddles at 75 rpm, and aliquots each of 2 mL were withdrawn at different time intervals (0.25, 0.5, 1, 2, 3, 4 and 5 h). Withdrawn samples were replaced by equal volumes of fresh release medium [28]. The samples were assayed by the HPLC method as described above.

#### Mathematical Modeling of the Drug Release

To explain the drug release mechanism, data obtained from release tests were studied under different mathematical models [29,30,31].

Zero order kinetic:

F = k × t,

first order kinetic:

lnF = k × t,

Higuchi model:

F = k × t^1/2^,

Korsmeyer–Peppas model:

F = k × t^n^,

where F represents the fraction of released drug, k is the constant connected with the release and t is the time.

### 2.6. Antimicrobial Activity

The antibacterial activity of designed gel formulations was evaluated by the plate diffusion method on Muller–Hinton agar. Tested microorganisms included the Gram(+) bacteria *Staphylococcus aureus* ATCC 29213 and the Gram(−) bacteria *Escherichia coli* ATCC 25922. Broth cultures (100 µL), adjusted to yield approximately 10^7^ CFU/mL (density corresponding to 0.5 McFarland scale), were inoculated on plates containing Mueller–Hinton medium. After 15 min of drying at room temperature, wells with a diameter of 5 mm were cut out in agar plates, into which prepared gel formulations (100 mg) were placed. The plates were incubated at 37 °C for 24 h. The results were recorded by measuring the zones of growth inhibition surrounding the wells using a caliper (Mitutoyo, Kawasaki, Japan) with an accuracy of 0.1 mm [9,23]. Hydrogel, oleogel and bigel placebo, and MET, MMET and the commercially available product Metronidazol 100 mg/g (C) were used as controls.

### 2.7. Ex Vivo Permeation Study

Gels containing MMET were selected for the ex vivo permeation study due to their better release profile and antibacterial activity. Permeation studies were accompanied in a flow through cell system equipped with thermostatic Teflon Bronaugh diffusion chambers and a peristaltic pump (MCP Process IP65, Ismatec). Porcine buccal mucosa from large white pigs weighting ~200 kg were from the veterinary service of a local slaughterhouse (Turośń Kościelna, Poland). Freshly excised tissue was preserved in the isotonic saline solution and kept at −20 °C for no longer than 60 days. Prior to experiments, tissue was thawed at room temperature and cut into pieces (weight 80–100 mg). In brief, porcine buccal mucosa was sandwiched between the two compartments with the mucosal side facing the donor compartment and equilibrated with phosphate buffer solution (pH 6.8) for 1 h. The diffusion area of the tissue was 0.81 cm^2^. After the equilibration period, a proper amount of tested formulations with 50 mg of MMET was carefully applied over the tissue surface and protected with parafilm. Commercially available ointment with MET was used as a control. The acceptor medium (phosphate buffer with addition of 0.005% of sodium azide) was then recirculated beneath the buccal mucosa at a constant rate of 30 mL/h. The sink conditions were confirmed during preliminary studies. At predetermined time intervals, samples of acceptor medium were collected, filtered through CA 0.45 µm filters and analyzed for drug content using the HPLC method. The samples were replaced by the same volume of acceptor solution to assure sink conditions.

At the end of the studies, the suspension from the donor compartment was carefully aspirated to a flat-bottom flask and washed with simulated saliva solution (SSS, pH 6.8) and methanol until complete MMET removal from the top of the tissue. The drug retained in the buccal mucosa was extracted by methanol at 30 °C for 4 h. After having been filtered through 0.45 µm nylon filters, the extract was analyzed for the amount of drug retained in the human vaginal epithelium by the HPLC method. Passive mucosal permeability and retention behavior were expressed as the amount of drug per unit area of the tissue [32,33].

### 2.8. Statistical Analysis

Results are presented as the mean ± standard deviation (SD) based on six independent experiments. Statistical analysis was done by one-way analysis of variance (ANOVA) using Statistica 10.0 software (StatSoft, Kraków, Poland). A probability level of *P* < 0.05 was considered as significant.

## 3. Results and Discussion

### 3.1. Micronization of MET

Micronization by the milling technique is a conventional method for particle size reduction and is frequently used for improving the solubility of drugs. This process does not enhance the equilibrium solubility of the drug itself, but it raises the dissolution rate by decreasing their size and increasing the surface area of the active compound [34,35]. Different milling parameters such as time and frequency were tested to choose the best conditions of this process. Settings were selected based on solubility testing and microscopic observation of particles size. The results of this studies are revealed in Table 2.

**Figure 1:**
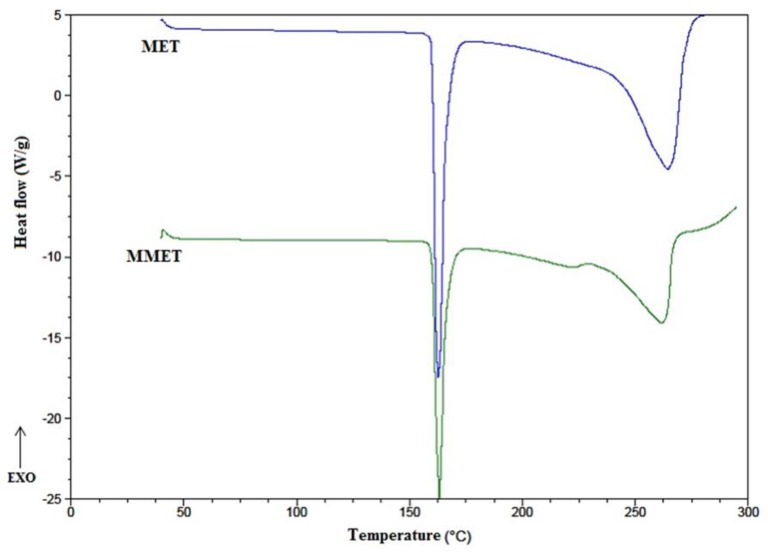
DSC thermographs of unmilled metronidazole (MET) and metronidazole milled for 30 min at 20 Hz (MMET).

As the most appropriate milling process conditions, 30 min at 20 Hz were considered. The ball milling technique increased the solubility of MET, especially in the case of solubility in linseed oil. In addition, homogeneous particle sizes of approximately 10 µm were obtained. Many compounds may undergo polymorphic changes during different technological processes, including milling. For this reason, the effect of milling on the crystal transformation of MET was investigated. DSC (Differential Scanning Calorimetry) is a thermoanalytical technique that can provide information regarding polymorphic changes in the milled material. It was found that the milling process under the chosen conditions (30 min at 20 Hz) did not influence MET crystallinity. The substance retained its properties, as confirmed by the DSC thermograph (Figure 1). As is visible in the DSC curve, the endothermic peak of MET and micronized metronidazole (MMET) showed no differences.

### 3.2. Preparation and Physicochemical Characteristics of Hydrogels, Oleogels and Bigels

During the design of semi-solid formulations for administration to the oral mucosa, the choice of appropriate vehicle and the selection of adequate excipients play essential roles. They influence the quality of the dosage form, its stability, drug release and efficacy. The choice of an optimal vehicle depends on many factors, such as the characteristic of the active substance, required therapeutic activity and stability of the final product. The appropriate vehicle should be smooth, inert, odorless, tasteless, physically and chemically stable and compatible with the active ingredient. It should neither irritate nor sensitize the oral mucosa. Moreover, it should be of such a consistency that spreads easily when stress force is applied and should also remain in the application site for a long time [36]. In gel formulations, various polymeric materials are used to obtain a viscous gel with a network structure. Polymers have played important roles in the preparation of pharmaceutical semi-solid products. They are used as rheology modifiers commonly referred to as thickeners or viscosifiers and are present in most products. Thickeners come from both natural and synthetic sources [37]. In our study we used two types of gelling agents, namely colloidal silicon dioxide and sodium alginate. Colloidal silicon dioxide (Aerosil^®^, fumed silica) has been successfully used to increase viscosity and impart thixotropic character to lipophilic ointments and non-aqueous suspensions and create oleogels for pharmaceutical use. These effects are due to the formation of a three-dimensional network between the silica particles via hydrogen bonding, thus imparting a paste-like consistency, especially with oils, depending on the polarity of the oil [38,39]. Alginate is a naturally derived polysaccharide that has been widely used to obtain different types of drug carriers. It is composed of (1–4)β-d-mannuronic acid and α-l-guluronic acid residues linked either randomly or as homopolymeric blocks. Sodium alginate has been commonly used as a gelling agent for hydrogels. Alginate hydrogels are considered for topical formulations, wound healing preparations or local delivery of drugs targeting oral cavity. They are characterized by pseudotropic behavior, biocompatibility, low toxicity and nonimmunogenicity [40].

The prepared gel formulations with MET and MMET were inspected visually for their color, homogeneity, consistency and phase separation. Hydrogels and oleogels were white or yellow (as a result of linseed oil used in the formulation). Formulations showed good homogeneity, smooth consistency with the absence of syneresis and they were easily spreadable. MET or MMET were evenly suspended in the bases. Stable and homogeneous bigels (formulations composed of sodium alginate hydrogel and linseed oil oleogel) were obtained only using the hydrogel to oleogel weight ratio of 70:30. Prepared bigels were smooth, homogenous, slightly yellow in color, creamy in appearance and they were not greasy in touch. In bigels formulated with a 50:50 weight ratio, syneresis (phase separation) 24 h after preparation was observed. In the case when a 30:70 proportion of hydrogel to oleogel was used, the consistency of the bigel was too firm and it was characterized by poor spreading.

The average content of active substance was in the acceptable USP pharmacopoeial limit (90%–110% of the labeled amount) for semisolid formulations containing metronidazole [41]. The range was respectively labeled as 92.3% to 102% for formulations with MET and 95.4% to 99.9% for gels with MMET (Table 3), which might indicate that the drug compound during the preparation process was uniformly dispersed and did not degrade.

The pH of a product may influence the solubility and stability of an active ingredient in the formulation. The pH values of prepared formulations with MET and MMET were in the range from 6.0 to 8.9, and the lowest values were observed in the case of oleogels (Table 3). Moreover, placebo hydrogel and bigel were characterized by slightly higher pH than preparations containing the active substance. In contrast, an inverse relationship was observed in the case of oleogels. The most appropriate pH when considering the substance stability (pH approximately 5.6) [42] was obtained for oleogels.

**Figure 2:**
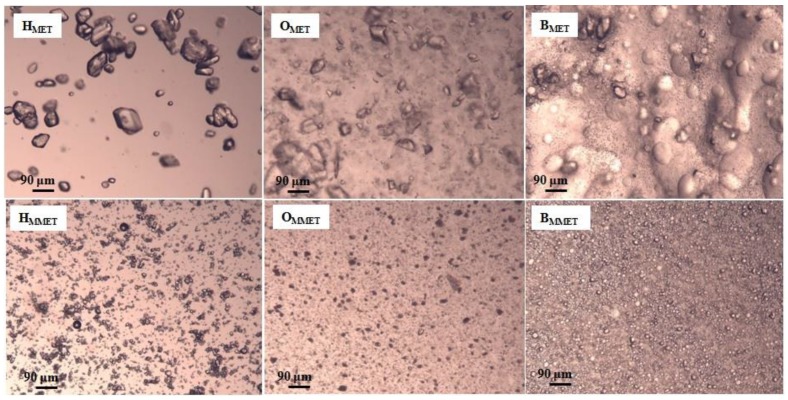
Microscopic images of prepared hydrogels, oleogels and bigels containing MET (HMET, OMET, BMET) or MMET (HMMET, OMMET, BMMET) under magnification 100×.

Significant differences in the size of suspended particles were observed (Table 3, Figure 2). In preparations containing micronized metronidazole (MMET), the average particle size was about 19 µm; they possessed a similar size and they were evenly dispersed. However, in the case of non-micronized MET, the particles were much larger than 90 μm and were on average as much as 139 µm [43]. The size of dispersed particles is important for application reasons; moreover, it may influence the release of the active substance from the semisolid dosage forms and affect the penetration through membranes. The active substance should be uniformly distributed in the vehicle, and the particles should be of appropriate size, because smaller particles dissolve faster, which determines better absorption. Large particles may be perceptible during application and cause unpleasant graininess.

**Figure 3:**
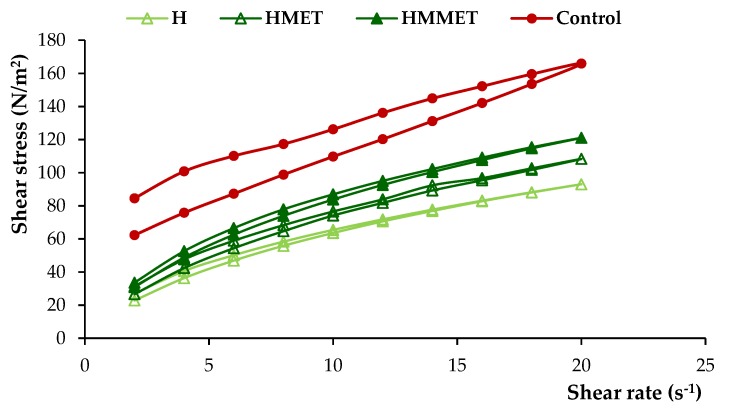

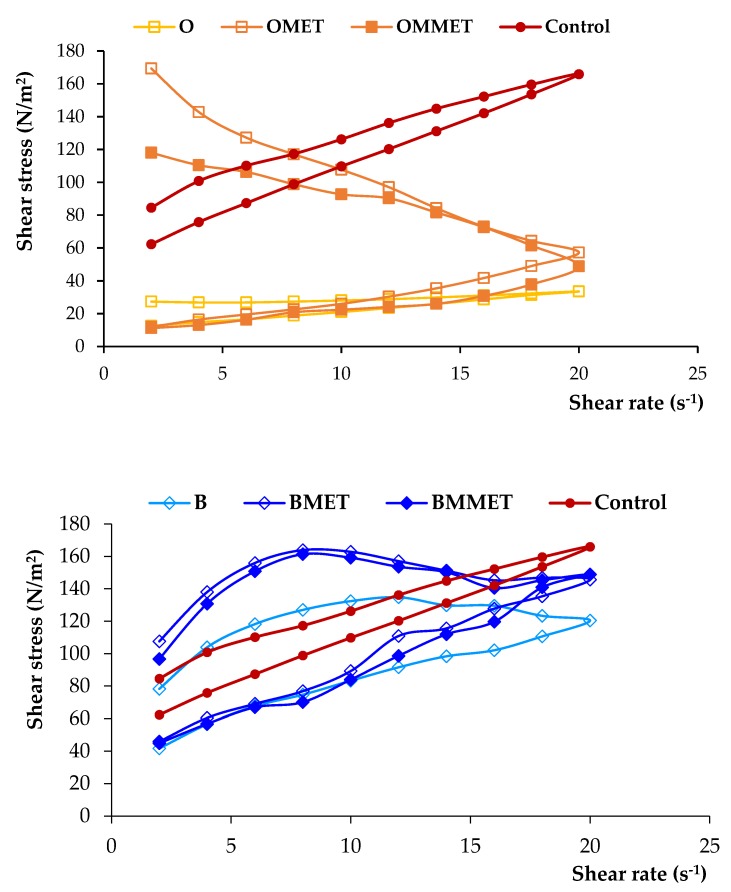
Rheograms of hydrogels, oleogels and bigels containing MET (HMET, OMET, BMET) or MMET (HMMET, OMMET, BMMET), placebo gels (H, O, B) and commercially available product (C).

The dosage form designed for local administration to the oral mucosa should be characterized by proper viscosity to provide semi-solid consistency, which will guarantee good spreadability and enable sufficiently long contact time with the mucosa. It may also influence the textural properties (firmness, compressibility, adhesiveness) and the release of the drug. Prepared gel formulations were found to have significantly different viscosity values (ranging from 2798 to 13,236 mPa∙s, Table 3). The highest viscosity was noticed in the case of bigel, and the lowest viscosity was possessed by oleogel. A high viscosity value of the preparation gives the possibility of extended contact with the mucosa, which is particularly important for formulations containing antimicrobial substances applied locally. The addition of metronidazole increased viscosity values, which was especially noticeable in oleogel formulations. Rheological characterization is crucial to understanding the essential nature of a system, study the effect of different parameters on the product quality and predict changes occurring upon storage or even for patient approval. Figure 3 demonstrates the rheograms of prepared hydrogels, oleogels, bigels with MET or MMET and control formulations.

From the rheograms it was found that all formulations were non-Newtonian, pseudoplastic systems and they exhibited a shear-thinning behavior. The shear-thinning phenomenon is considered a relevant parameter for local application of the semisolid formulation. This facilitates the formation of a thin layer of the gel preparation when it is locally applied, which results in the efficient delivery of active agents at the site of application. Moreover, designed gels possessed thixotropic properties, as evidenced by the hysteresis loops noticeable on rheograms. A thixotropic system reveals loss in apparent viscosity over time at a constant shear rate, while the shear stress is removed. Subsequently, the apparent viscosity constantly increases and returns to its primary value. It is assumed that under shearing stress, the bonding between polymers chains are broken down, resulting in a more fluid-like low viscosity system, while in the absence of mechanical forces, polymer particles rejoin again, and the three-dimensional network rebuilds, restoring the primary structure of the material [27].

Texture can be regarded as a manifestation of the rheological properties of a product. Texture profile analysis provides information on the response to the external force. It is valuable to predict sample behavior under physiological conditions, such as the application of force during sample administration, and to verify the ease of semi-solid formulation removal from the container or its spreadability at the site of application. An ideal semi-solid formulation for use to the oral mucosa should possess properties that enable easy application and spreading, determined by adequate firmness and compressibility. Additionally, it is important to achieve prolonged duration at the application site without the structural damage that can be provided by higher values of adhesiveness. Firmness is described as the maximal force required to attain a given deformation. Compressibility (calculated from the positive area under the force–time curve) defines the work required to deform the product during compression of the probe, and it is correlated with the spreadability on the oral mucosa. Firmness and compressibility quantify sample deformation under compression and shear. They are related to sample consistency. Low values of firmness and compressibility ensure that minimum work is required to remove the formulation from the container and to administer it at the site of application. Adhesiveness is the negative area covered by the force–time curve, and its value represents the work required to remove the probe from the sample, which is connected with the breaking of cohesive bonds. In the case of semi-solid formulations for use on the oral mucosa, higher values are more favorable to ensure prolonged adhesion [26,27,28].

Placebo formulations possessed slightly lower firmness, compressibility and adhesiveness compared with gel formulations containing MET (Table 4). The addition of active substance increased the measured parameters, which was particularly noticeable in the case of oleogels and bigels. The highest values of mechanical parameters were recorded in the case of oleogel (OMET, OMMET), while the lowest were for hydrogels (HMET, HMMET).

### 3.3. Ex Vivo Bioadhesive Properties

Bioadhesion refers to the adhesive interaction between the polymeric drug delivery system and the mucus covered oral cavity. If the adhesion is on a mucosal surface, then the phenomenon is called mucoadhesion. Strong adhesion can occur if two surfaces are able to form either covalent or ionic bonds. Additionally, weaker forces, such as polar (dipole–dipole), hydrogen bonding or van der Waals interactions are involved in bonding the two surfaces [44]. Difficulty connecting with the semi-solid drug carriers used as local delivery systems targeting oral cavity indicates their poor retention at the site of application. This can be caused by its limited interaction with the oral mucosa, mechanical removal by the saliva and applied stress during chewing and swallowing. The bioadhesive properties of the polymers used to formulate gel drug delivery systems provide these carriers to adhere to the oral mucosa and in consequence extend the retention time of the dosage form at the site of application.

**Figure 4:**
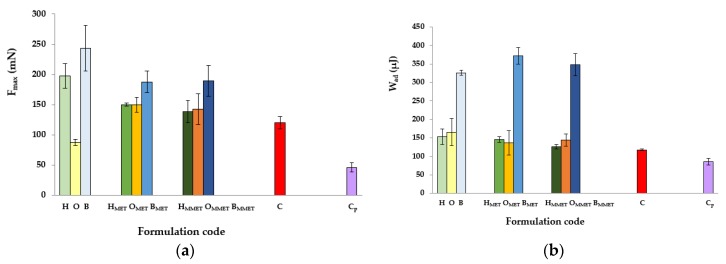
Ex vivo bioadhesive properties of hydrogels, oleogels and bigels containing MET (HMET, OMET, BMET) or MMET (HMMET, OMMET, BMMET), placebo gels (H, O, B) and controls; commercially available product (C) and cellulose paper (C_p_) determined as the maximum detachment force F_max_ (**a**) and the work of adhesion W_ad_ (**b**).

It was observed that all formulations were characterized by bioadhesive properties, which might be explained by the formation of hydrogen bonds between the proteins present in the mucosa and the polymer chains used as gelators. The results of the ex vivo study carried out using the porcine buccal mucosa as a model adhesive layer are presented in Figure 4a,b.

The greatest values of F_max_ and W_ad_ were noticed in the case of bigels, characterized by high viscosity and high values of mechanical parameters. This is in accordance with other authors who showed a correlation between rheological and textural results, that is, an increase in the interaction between a highly viscous sample and mucus [27,45,46]. Formulations that show high viscosity have demonstrated greater resistance to elution and, consequently, they remain longer at the action site [47]. Furthermore, bigels are hybrid gels, and the composition of the systems allows for significant formation of hydrogen bonds mediated by the collective interaction of non-polar and polar constituents of the gel matrix, including sodium alginate (polymer characterized by unique swelling, gelling and mucoadhesive properties) and silica dioxide, thus increasing viscosity and imparting bioadhesive properties [48,49]. On the other hand, the low bioadhesion may be explained on the basis of some studies that concluded that a more rigid polymer network may prevent the proteins present in the biological membrane from interacting with the polymer chain, decreasing the bioadhesive properties of the system [39]. Addition of drug reduced F_max_ values in bigels and hydrogels, while in the case of oleogels, significant growth of F_max_ was observed. Moreover, bigels containing drug were characterized by modestly higher W_ad_ compared to placebo, while for hydrogels and oleogels, a decrease in the examined value was noted. Higher values of bioadhesion parameters in case of some placebo gels compared with MET loaded formulations might indicate that active substance hinder polymer contact with the mucous membrane.

### 3.4. In Vitro Release

From the in vitro dissolution studies, it is possible to determine how the type and composition of the formulation affects drug release. Drug release from semi-solid preparations depends on several parameters such as the nature of the vehicle, its viscosity, solubility of the active compound in the vehicle and acceptor medium, as well as drug partition coefficient between the vehicle and water. The type of the semi-solid vehicle determines the ability of the acceptor fluid to penetrate into the formulation; it is considered that the release rate of hydrophilic and moderately hydrophilic active substances usually increases with hydrophilicity of the vehicle in the following order: hydrophobic base (anhydrous ointments, oleogels) < emulsion (mixtures of hydrophilic and hydrophobic phase creams w/o or o/w, bigels, emulgels) < hydrophilic base (hydrogels) [11].

**Figure 5:**
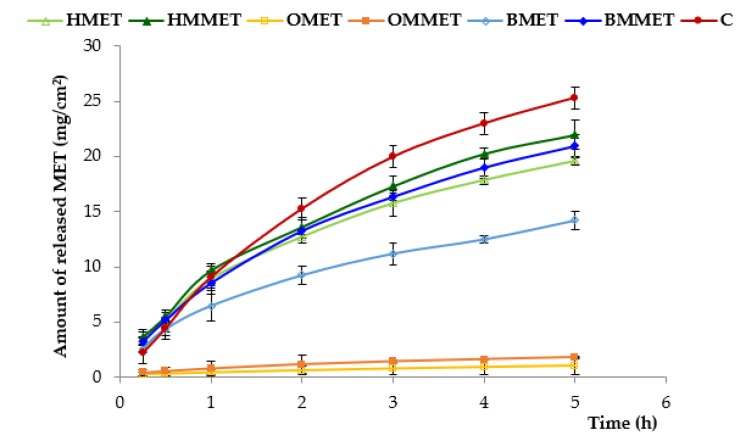
In vitro release from hydrogels, oleogels and bigels containing MET (HMET, OMET, BMET) or MMET (HMMET, OMMET, BMMET) and commercially available product (C).

The release of MET from different types of gel formulations is demonstrated in Figure 5.

It was shown that the amount of released MET from oleogels was significantly the lowest and it can be ranked in the following descending order: C (control formulation, marked product) > HMMET > HMET ≈ BMMET > BMET > OMMET > OMET. The largest amount of released MET was observed for the control formulation (ointment base on PEG) and hydrogel containing micronized substance (HMMET); after 5 h, the cumulative amount of released metronidazole was, respectively, 25 mg/cm^2^ and 22 mg/cm^2^. Moreover, these results are due to the increase of hydrophilicity of formulations that facilitated the penetration of the release medium into the vehicle and promoted diffusion of the active substance. Oleogels demonstrated the slightest drug release (after 5 h, cumulative amount of released MET and MMET was only 1 mg/cm^2^ and 1.8 mg/cm^2^, respectively). The release rate from hydrophobic oleogels was slower than from hydrogels or bigels, owing to the partitioning of the MET between aqueous (release medium) and oil phase (oleogel). It should be also noted that micronization process improved release of MET for all prepared gel formulations, which was particularly evident in the case of bigel.

Mathematical models play a crucial role in the interpretation of the mechanism of drug release from a dosage form and aid understanding of the drug release kinetics. In order to better characterize the drug release behavior, the mechanism of drug release from designed gel formulations was analyzed according to various mathematical models, namely zero order kinetic, first order kinetic, Higuchi model and Korsmeyer–Peppas. The correlation coefficient (R^2^) and the values of the release exponent (n) were used to determine the best fit model. Zero-order kinetic describes the drug release rate from dosage forms independent of the drug concentration. First-order kinetic illustrates the dependency on the drug concentration i.e., when the drug concentration is high, the release rate is faster. In the case of Higuchi and Korsmeyer–Peppas models, release rate is variable in time, and these models are founded on the assumption of the accordance of the release with Fick’s law [29,30,31].

The kinetic model describing the process of MET or MMET release from prepared gel formulations was selected according to the highest correlation coefficient value (R^2^). The most fitted model to explain the drug release from all designed preparations was the Higuchi equation (Table 5).

The Higuchi kinetic plots were found to be fairly linear, as indicated by their highest regression values, and for all prepared formulations correlation coefficients values (R^2^) were in the range of 0.992–0.999. Higuchi’s model expresses diffusion of the drug in accordance with Fick’s law, where the rate of diffusion through the membrane is affected by the difference in drug concentrations across the membrane.

An important parameter describing the drug release is also the diffusion exponent n, the value of which could be used to characterize the release mechanisms. If n ≤ 0.5, the drug is released via Fickian difusion; when n = 1.0, the release takes place with zero-order kinetics. In the case where the value of the exponent n is in the range of 0.5 < n < 1.0, the release is indicated by anomalous transport governed by both above-mentioned processes [30,31]. Exponents for drug diffusion from prepared gel formulations were less than 0.5, which indicates that its release took place by diffusion in accordance with Fick’s law. In contrast, in the case of control preparations, the exponent n was 0.607, which implies an anomalous or non-Fickian diffusion.

### 3.5. Antimicrobial Activity

**Figure 6:**
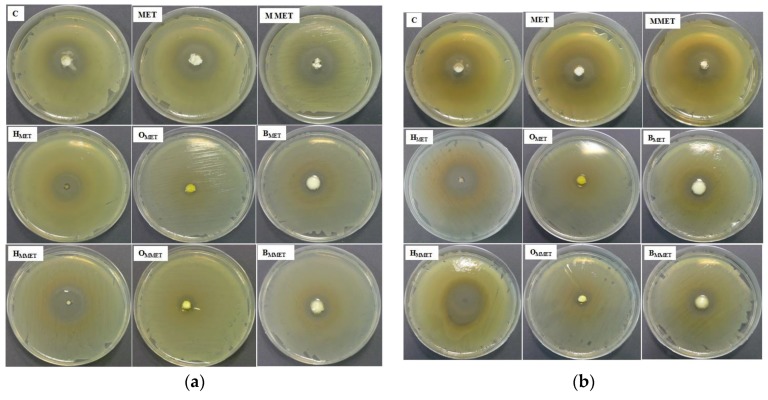
Antibacterial activity against *S. aureus* (**a**) and *E. coli* (**b**) of prepared hydrogels, oleogels, bigels containing MET (HMET, OMET, BMET), MMET (HMMET, OMMET, BMMET) and pure MET, MMET, commercially available product (C) as controls.

In order to estimate the therapeutic efficacy of different types of gel formulations, their antimicrobial activity was estimated (Table 6, Figure 6).

It was found that the highest antibacterial activity against both *S. aureus* and *E. coli* was observed in the case of hydrogels. Bigels possessed antibacterial properties similar to the commercially available product. Lower antibacterial activity was noticed in the case of oleogels. This might be due to the relatively weak diffusion of MET from lipophilic base types of oleogels. Moreover, drug micronization improved antimicrobial properties against tested strains in all formulations. The blank gels did not show any antimicrobial activity (data not shown).

### 3.6. Ex Vivo Permeation Study

**Figure 7:**
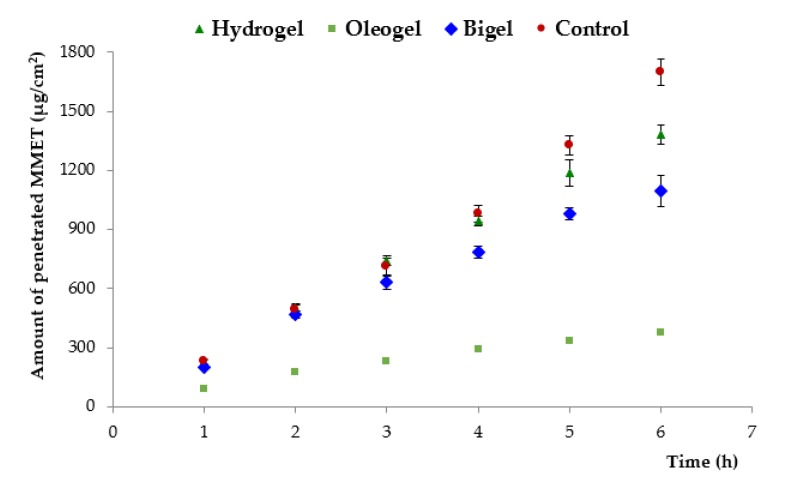
Penetration of MMET from hydrogel, oleogel and bigel formulations as compared to commercially available ointment with MET through porcine buccal mucosa (expressed as the amount of drug penetrated per unit of tissue area to acceptor medium).

The drug permeation profile from tested semi-solid formulations and commercially available product across an excised porcine buccal mucosa is presented in Figure 7. Porcine buccal mucosa was selected due to its similarity with human buccal tissue in terms of permeability, structural organization and barrier lipid composition [50]. To obtain a maximal penetration rate, MMET was applied in infinite dose. The variance in pH among formulations (pH 6.0–8.0, Table 3) had no influence on MMET ability to permeate across cell membranes as the drug was in a non-ionized state (pKa 2.5) regardless of tested preparation.

With regard to oral mucosa drug delivery, sufficiently long residence time is crucial for drug permeability and pharmacological efficacy. Longer residence time is usually limited because of the washing effect of saliva. The total amount of drug penetrated across the excised buccal mucosa to the receptor medium within the first hour of incubation varied between 73 and 190 µg/cm^2^, which may be confirmed by the high mucosal permeability of MMET, as reported in the literature [19]. Basically, the permeation efflux of MMET from commercially available product was markedly higher (370 µg/cm^2^/h) than from all other tested formulations, which most probably resulted from the presence of PEG acting as penetration enhancer and solubilizer. Interestingly, the bigel formulation with 30% lipid component (considered as a factor limiting drug solubility and in consequence its absorption), displayed high initial drug penetration rate. Moreover, despite higher viscosity and more firmness structure (Table 3 and Table 4), the maximum permeation coefficient observed for bigel in 2 h of incubation (270 µg/cm^2^/h) was comparable to the value obtained for hydrogel (280 µg/cm^2^/h), suggesting enhanced diffusion of drug through the bigel matrix. Significant differences in MMET permeation from tested formulations were observed after 5 h. Hydrogel exhibited a higher level of cumulative permeation (approximately 1200 µg/cm^2^ after 5 h of incubation), whereas significantly lower permeation of MMET through mucosal tissue was observed for the oleogel preparation (about 330 µg/cm^2^). This observation may be attributed to profound differences in drug solubility in aqueous media and linseed oil (Table 1).

**Figure 8:**
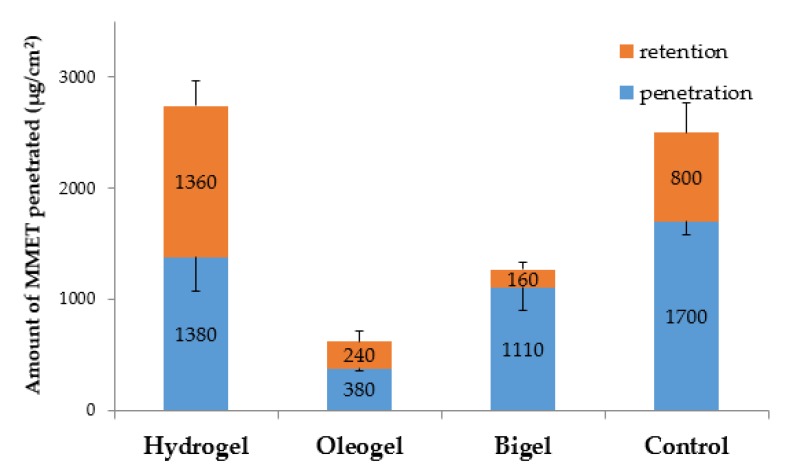
Ex vivo cumulative penetration and tissue retention of MMET from hydrogel, oleogel and bigel formulations as compared to commercially available ointment with MET in porcine buccal mucosa (expressed as the amount of drug penetrated per unit of tissue area).

The impact of the type and composition of formulation on MMET retention behavior in mucosal tissue was also noticed (Figure 8).

The retention in porcine buccal mucosa varied in a broad range between 160 µg/cm^2^ and 1360 µg/cm^2^ after 6 h of incubation. A significant increase in drug retention was noted after tissue incubation with the hydrogel formulation as compared to bigel and oleogel preparations. An enhanced mucosal retention may favor prolonged drug activity at the application site, but on the other hand it might correlate with the cytotoxic effect on the mucosal cells [51,52].

## 4. Conclusions

Mucoadhesive gels systems for periodontal diseases offer many advantages over other conventional preparations used in this treatment. They are characterized by better efficacy for a longer period because they remain at the application site and are more difficult to be removed by the saliva. Based on obtained results, it can be concluded that the designed gel formulations exhibited acceptable physicochemical features in terms of pH, drug content, viscosity and textural properties. Additionally, micronization of MET with the use of the ball milling technique allowed adequate homogeneous particle sizes to be obtained. It also positively influenced the release of MET from gel formulations and their antimicrobial efficiency. The bigels, hydrogels and oleogels obtained were non-Newtonian systems, showing a shear-thinning behavior with thixotropic properties. Moreover, it was noticed that all formulations were characterized by mucoadhesive properties. It was demonstrated that the viscosity, mechanical features, bioadhesive properties, antimicrobial activity, in vitro drug release and ex vivo permeation of MET were influenced by the type of gel formulations. The results of the in vitro release study indicated that the hydrophobic formulation (oleogel) showed lower drug release rate than the hydrophilic (hydrogel) and the mixture of hydrophilic and hydrophobic bases (bigel). It was found that higher antibacterial efficiency against tested strains was observed in the case of hydrogels and bigels. An ex vivo permeation study using porcine buccal mucosa as a model membrane demonstrated that bigel formulation was characterized by high initial permeability rate to assure fast therapeutic effect with simultaneous moderate retention in mucosal tissue to decrease the risk of local cytotoxicity. Results of the study showed that the tested bigel containing micronized drug seemed to be a promising and effective form for local delivery of metronidazole.

## Figures and Tables

**Table 1:** Compositions of designed placebo hydrogel (H), oleogel (O), bigel (B), hydrogels, oleogels and bigels containing unprocessed MET (HMET, OMET, BMET) or micronized MMET (HMMET, OMMET, BMMET).

Ingredient (g)	Hydrogels (H)			Oleogels (O)			Bigels (B) (H+O 70:30)		
	H	HMET	HMMET	O	OMET	OMMET	B	BMET	BMMET
MET	-	10.0	-	-	10.0	-	-	10.0	-
MMET	-	-	10.0	-	-	10.0	-	-	10.0
Aerosil^®^200	-	-	-	5.0	5.0	5.0	1.5	1.5	1.5
Tween 80	-	-	-	1.0	1.0	1.0	0.3	0.3	0.3
Tocopheryl acetate	-	-	-	0.05	0.05	0.05	0.05	0.05	0.05
Linseed oil	-	-	-	up to 100.0	up to 100.0	up to 100.0	up to 30.0	up to 30.0	up to 30.0
Sodium alginate	1.75	1.75	1.75	-	-	-	1.23	1.23	1.23
Glycerol 85%	20.0	20.0	20.0	-	-	-	14.0	14.0	14.0
Ethyl alcohol 96%	10.0	10.0	10.0	-	-	-	7.0	7.0	7.0
Methyl paraben	0.2	0.2	0.2	-	-	-	0.2	0.2	0.2
Propyl paraben	0.02	0.02	0.02	-	-	-	0.02	0.02	0.02
Purified water	up to 100	up to 100	up to 100	-	-	-	up to 70.0	up to 70.0	up to 70.0

**Table 2:** Particles size and solubility in various solvents of unmilled metronidazole (MET) and metronidazole milled for 30 min at 20 Hz (MMET).

Ball Milling Parameters	Solubility in Water (mg/mL)	Solubility in Phosphate Buffer pH 6.8 (mg/mL)	Solubility in Linseed Oil (mg/mL)	Particles Size (µm)
-	7.98 ± 1.65	6.91 ± 3.08	0.363 ± 7.34	334.6 ± 384.6
5 min/20 Hz	8.52 ± 2.18	6.91 ± 1.50	0.382 ± 6.89	33.9 ± 33.6
10 min/20 Hz	8.77 ± 3.78	7.39 ± 2.58	0.408 ± 3.19	15.2 ± 6.4
20 min/20 Hz	8.92 ± 3.05	7.49 ± 2.69	0.444 ± 4.56	10.7 ± 3.9
30 min/20 Hz	10.00 ± 2.22	8.29 ± 2.78	0.445 ± 6.98	10.7 ± 3.8
60 min/20 Hz	10.04 ± 1.60	9.03 ± 3.09	0.445 ± 9.24	10.4 ± 4.1

**Table 3:** Drug content, pH, particles size and viscosity of prepared hydrogels, oleogels and bigels containing MET (HMET, OMET, BMET) or MMET (HMMET, OMMET, BMMET), placebo gels (H, O, B) and commercially available product (C).

Formulation Code	Drug Content (%)	pH	Particles Size (µm)	Viscosity * (mPa∙s)
C	100.8 ± 0.3	9.5 ± 0.02	74.7 ± 31.4	12,621 ± 147
H	-	9.5 ± 0.01	-	6542 ± 61
HMET	102.0 ± 6.4	8.9 ± 0.01	138.4 ± 66.7	8563 ± 73
HMMET	99.9 ± 1.0	8.6 ± 0.02	18.9 ± 8.7	7660 ± 42
O	-	5.9 ± 0.02	-	2798 ± 20
OMET	92.3 ± 2.3	6.4 ± 0.01	101.1 ± 67.4	10,769 ± 98
OMMET	95.4 ± 0.2	6.0 ± 0.01	18.5 ± 8.5	9267 ± 32
B	-	9.0 ± 0.02	-	13,236 ± 182
BMET	98.6 ± 2.2	8.5 ± 0.01	103.3 ± 41.4	16,279 ± 148
BMMET	97.6 ± 3.9	8.1 ± 0.01	18.6 ± 9.2	14,220 ± 60

* viscosity was measured at the shear rate 10.00 s^−1^ at 25 °C.

**Table 4:** Textural properties of bigels, hydrogels, oleogels containing MET (HMET, OMET, BMET) or MMET (HMMET, OMMET, BMMET), placebo gels (H, O, B) and commercially available product (C).

Formulation Code	Firmness (g)	Compressibility (g·s)	Adhesiveness (g·s)
C	247.0 ± 16.8	267.3 ± 26.0	−352.6 ± 16.9
H	54.7 ± 3.0	110.0 ± 4.8	−135.6 ± 2.4
HMET	55.2 ± 3.9	113.6 ± 7.3	−139.3 ± 8.1
HMMET	55.0 ± 4.4	110.2 ± 3.6	−137.3 ± 6.5
O	25.7 ± 0.5	55.5 ± 0.8	−58.8 ± 0.7
OMET	251.6 ± 2.6	386.5 ± 23.3	−369.0 ± 35.7
OMMET	229.3 ± 6.8	326.1 ± 4.1	−344.6 ± 23.5
B	112.5 ± 8.3	214.8 ± 17.8	−248.2 ± 17.0
BMET	136.6 ± 11.7	251.1 ± 29.5	−315.8 ± 15.5
BMMET	127.5 ± 2.7	240.7 ± 6.7	−273.6 ± 5.8

**Table 5:** Models of MET/MMET release from designed formulations and commercially available product (C).

Formulation Code	Kinetic Models								
	Zero Order Kinetics		First Order Kinetics		Higuchi Model		Korsmeyer–Peppas Model		
	R^2^	K_0_	R^2^	K_I_	R^2^	K_H_	R^2^	K_KP_	n
C	0.961	9.257	0.985	0.130	0.998	26.012	0.908	0.484	0.607
HMET	0.980	5.428	0.988	0.092	0.999	19.555	0.953	0.722	0.472
HMMET	0.979	7.078	0.993	0.102	0.998	20.942	0.935	0.730	0.481
OMET	0.981	0.301	0.986	0.003	0.992	0.827	0.988	0.604	0.334
OMMET	0.969	0.547	0.970	0.006	0.999	1.532	0.960	0.673	0.374
BMET	0.969	6.989	0.985	0.067	0.998	15.061	0.946	0.682	0.454
BMMET	0.959	7.037	0.979	0.094	0.998	19.797	0.951	0.721	0.472

**Table 6:** Antibacterial activity of prepared hydrogels, oleogels, bigels containing MET (HMET, OMET, BMET), MMET (HMMET, OMMET, BMMET) and pure MET, MMET, commercially available product (C) as controls.

Zone of Inhibition (mm)	Name of the Strain	
	*S. aureus* ATCC 29213	*E. coli* ATCC 25922
C	18.7 ± 0.6	20.7 ± 0.6
MET	21.7 ± 1.2	22.0 ± 0.0
MMET	22.3 ± 0.6	24.3 ± 0.6
H	0.0 ± 0.0	0.0 ± 0.0
HMET	21.0 ± 1.0	22.3 ± 0.6
HMMET	21.7 ± 0.6	24.0 ± 0.0
O	0.0 ± 0.0	0.0 ± 0.0
OMET	8.0 ± 0.0	13.3 ± 1.5
OMMET	9.3 ± 1.2	15.0 ± 1.0
B	0.0 ± 0.0	0.0 ± 0.0
BMET	18.7 ± 1.2	21.0 ± 0.0
BMMET	19.3 ± 1.2	22.7 ± 1.2
